# Mutual interaction of red blood cells influenced by nanoparticles

**DOI:** 10.1038/s41598-019-41643-x

**Published:** 2019-03-26

**Authors:** Tatiana Avsievich, Alexey Popov, Alexander Bykov, Igor Meglinski

**Affiliations:** 1 Opto-Electronics and Measurement Techniques, University of Oulu, P.O. Box 4500, Oulu, 90014 Finland; 2 Interdisciplinary Laboratory of Biophotonics, National Research Tomsk State University, Tomsk, 634050 Russia; 3 National Research Nuclear University – MEPhI, Institute of Engineering Physics for Biomedicine (PhysBio), Moscow, 115409 Russia

**Keywords:** Optical manipulation and tweezers, Biomedical engineering

## Abstract

Despite extensive studies on different types of nanoparticles as potential drug carriers, the application of red blood cells (RBCs) as natural transport agents for systemic drug delivery is considered a new paradigm in modern medicine and possesses great potential. There is a lack of studies on the influence of drug carriers of different compositions on RBCs, especially regarding their potential impact on human health. Here, we apply conventional microscopy to observe the formation of RBC aggregates and optical tweezers to quantitatively assess the mutual interaction of RBCs incubated with inorganic and polymeric nanoparticles. Scanning electron microscopy is utilized for direct observation of nanoparticle localization on RBC membranes. The experiments are performed in a platelet-free blood plasma mimicking the RBC natural environment. We show that nanodiamonds influence mutual RBC interactions more antagonistically than other nanoparticles, resulting in higher aggregation forces and the formation of larger cell aggregates. In contrast, polymeric particles do not cause anomalous RBC aggregation. The results emphasize the application of optical tweezers for the direct quantitative assessment of the mutual interaction of RBCs influenced by nanomaterials.

## Introduction

Nanoparticles (NPs) are widely used in various applications, ranging from those in the pharmaceutical and cosmetic industries to modern medicine and biotechnologies^1^. The safety issues and efficacy testing of NPs used in biomedical applications are widely discussed elsewhere^2^. Although different types of NPs have been extensively used as drug carriers^3,4^, the application of red blood cells (RBCs) as natural transport agents for systemic drug delivery by placing NPs on the RBC surface is considered a new paradigm in modern medicine that possesses great potential^5^. In the frame of this paradigm, the interaction of nano- or sub-micro-particles with the RBC as a drug carrier has recently been studied in^6^. Two types of NPs, lysozyme-dextran (LDNG) and polystyrene (PSNP), were specifically tested for potential adverse and sensitizing effects^6^. Earlier, it was demonstrated that relatively large NP agglomerates (~0.2 μm) were able to penetrate RBCs through the membrane with no influence on the nature of the NP material^7^. Significant RBC agglutination and stronger adhesion caused by small NPs than larger ones were observed for different types of NPs^8–10^. Surface properties are considered one of the major factors for the hemocompatibility of NPs. In fact, hemocompatibility does not indicate any significant relationship between RBC adhesion and potential NP toxicity in blood vessels, as different physiopathological processes are linked to hemocompatibility^11^. While the adhesion of RBCs is often considered unfavourable for the elimination of NPs from the RBC surface (because such an adhesion hinders the clearance of RBCs from NPs), the actual mechanism of the influence of NPs on mutual RBC interaction is still unknown and requires clarification.

The adhesive interaction of RBCs has been extensively studied in the frame of cell-to-cell interactions induced by dextran macromolecules^12^, whereas data are lacking for native plasma solutions. While two hypotheses accounting for the cross-bridges^13^ and the depletion layer models^14^ are used to describe the mechanism of RBC interaction, they require experimental confirmation. The *bridging model* is based on the assumption that the interaction between adjacent RBCs is governed by fibrinogen macromolecules that form ‘cross-bridges’. In the *depletion model*, RBC aggregation is considered in terms of the formation of a polymer-free ‘depletion layer’ between RBCs, where osmotic pressure pushes the cells towards each other. The applicability of these models for the description of RBC interaction in native plasma (the cross-bridges model) and in macromolecule solutions (the depletion model) has been confirmed. However, in a mixture of macromolecules with similar sizes and concentrations proportional to those of proteins in blood plasma, the dependence of RBC adhesion tends to be closer to that observed in the cross-bridges model^15^.

Despite the importance of ultimately understanding the mutual interaction of RBCs influenced by different nanocomposites, there is a serious lack of comprehensive studies utilizing currently available imaging and diagnostic modalities. Conventional optical microscopy, scanning electron microscopy (with fixed cells) and transmission electron microscopy (with sections of fixed cells) are extensively used to assess the morphological properties of RBCs influenced by metal^16^, luminescent^17^, silicon^18^, graphene oxide^19^, and carbon NPs^20^. The dynamic light scattering (DLS) approach is promising for monitoring the shape of blood elements and their changes^21^ and is utilized extensively for NP characterization^22^.

Introduced by Arthur Ashkin, the experimental optical tweezer (OT) approach^23^ has been widely used for the optical trapping of particles and manipulation of single cells^24^, and recently, it has been applied to studies of mutual adhesive interaction of RBCs^25^. Here, we apply an OT experimental system developed in-house^26^ for the quantitative assessment of the mutual interaction of RBCs incubated with NPs of various types, whereas conventional optical microscopy and scanning electron microscopy (SEM) are used for direct observation of RBCs and NPs during their interaction.

## Results

**Figure 1:**
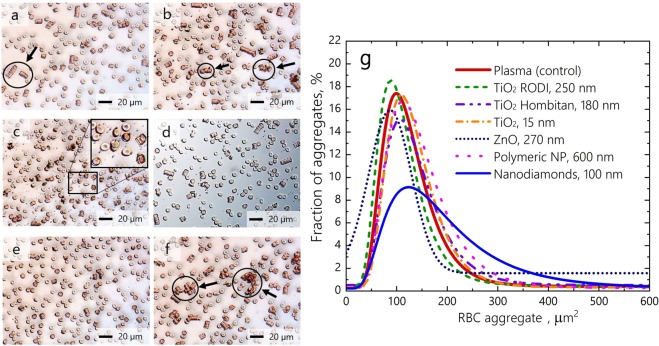
Relative size distribution of RBC aggregates observed by conventional optical microscopy under normal conditions and influencing by polymeric NP 600 nm (**a**), TiO_2_ RODI 250 nm NP (**b**), TiO_2_ Hombitan AN 180 nm NP (**c**), TiO_2_ 15 nm NP (**d**), ZnO 270 nm NP (**e**) and ND 100 nm (**f**). The RBC aggregates are encircled and indicated with arrows. (**g**) Distribution of RBC aggregates by occupied area based on the quantitative assessment of images in (**a–f**).

The conventional optical microscopy approach shows that RBCs incubated with different types of NP form aggregates of irregular size and shape that notably differ from those observed under normal conditions (Fig. 1). Under normal conditions and in the presence of polymeric NP, the RBCs typically form standard rouleaux-shaped aggregates (see Fig. 1a), whereas significant variations in both the shape and size of the RBC aggregates are observed when RBCs are incubated with different NPs (see Fig. 1b–f). It has also been found that the size of irregularly shaped aggregates becomes significantly larger (~hundreds of μm^2^) when RBCs are incubated with nanodiamonds (ND) (see Fig. 1f). Figure 1g shows the distribution of RBC aggregates by size quantitatively assessed utilizing conventional optical microscopy.

**Figure 2:**
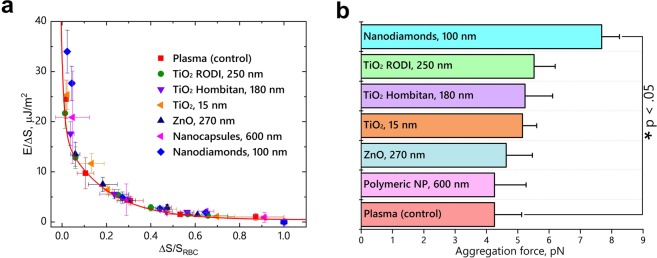
Energy of mutual RBC interaction influenced by NPs (**a**). The solid line fits the energy dependence obtained for RBCs in plasma (control sample) based on the cross-bridges model^27^. The aggregation forces of RBC interaction in plasma incubated with NPs (**b**). The U-test at p < 0.05 shows a significant difference in aggregation forces for the sample treated with NDs relative to the control. The details of the OT setup and the measurement procedures are presented in the Methods section.

The results of direct measurements of mutual cell-to-cell RBC interaction by the OT approach are presented in Fig. 2. The energy of the RBC interaction is normalized to the relative conjugated area ΔS^14^ (see Fig. 2a). The energy of mutual interaction of RBC is independent of the size and type of NP (see Fig. 2a) and agrees well with theoretical predictions obtained in the frame of the migration cross-bridges model^27^. In contrast, the aggregation forces *F*_*a*_ associated with the mutual interaction of RBC incubated with NP are increased compared to the interaction RBC forces in plasma or in the presence of polymeric NP. The values of aggregation forces obtained experimentally are *F*_*a*_ = 4.26 ± 0.87 pN (for the RBCs in plasma) and *F*_*a*_ = 4.60 ± 0.57 pN (for the RBCs incubated with polymeric NPs). The forces obtained for the other NPs (see Fig. 2b) were as follows: *F*_*a*_ = 4.63 ± 0.84 pN (RBCs with ZnO), *F*_*a*_ = 5.15 ± 0.45 pN (RBCs with TiO_2_ 15 nm), *F*_*a*_ = 5.23 ± 0.88 pN (RBCs with TiO_2_ Hombitan AN), *F*_*a*_ = 5.52 ± 0.68 (RBCs with TiO_2_ RODI), *F*_*a*_ = 7.68 ± 0.57 (RBCs with NDs).

Thus, NPs activate cell-to-cell interactions without strong binding to the membrane and causing a change in the spatial distribution of charge within the mediating NPs. The equilibrium configuration of membranes and mediating molecules appears to increase the adhesion forces between the membranes, whereas the total energy of mutual interaction does not change (see Fig. 2a). It is worth noting that the presence of polymeric NPs do not influence RBC interaction; the environment appears to be similar to that observed in blood plasma, including interaction forces and size distribution of RBC aggregates.

**Figure 3:**
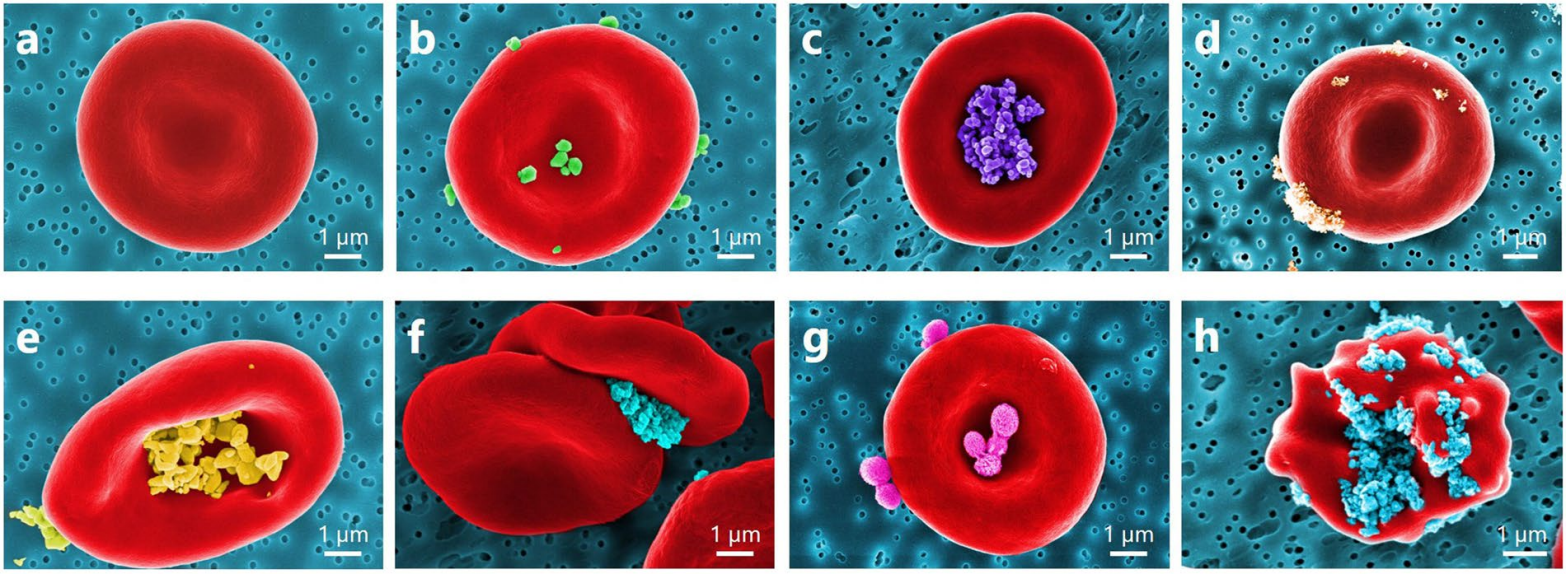
Coloured SEM images presenting a diversity of observed NP localizations on the RBC surface: (**a**) normal conditions; RBC incubated with (**b**) TiO_2_ RODI, (**c**) TiO_2_ Hombitan AN, (**d**) TiO_2_ 15 nm, (**e**) ZnO NPs, (**f**) NDs, and (**g**) polymeric NPs; (**h**) echinocyte form of RBC due to adhesion of NDs.

A direct observation of RBCs incubated with NP by SEM imaging shows a notable difference in the dispersion of NP localization on the surface of RBCs (Fig. 3a–h).

Despite the substantial difference in size, TiO_2_ RODI and TiO_2_ 15 nm NPs are distributed over the RBC surface (see, respectively, Fig. 3b,d). However, TiO_2_ Hombitan AN (see Fig. 3c) and ZnO (see Fig. 3e) NPs are localized in the central parts of RBC, but the influence of TiO_2_ Hombitan AN NPs on the aggregation forces is notably (approximately 10%) higher than that of the ZnO NPs (see Fig. 2b). The variations in size (range 15–250 nm) of TiO_2_-based NPs exhibit no influence on the aggregation of RBCs, as no changes compared to the control samples of RBCs in plasma are observed. Measured by OT, the adhesion of RBCs incubated with TiO_2_ and ZnO NPs of various sizes shows no difference in terms of interaction energy but demonstrates an increase of adhesion forces in the mutual interaction of RBCs. Thus, plasma proteins bound to the NP surface influence NP interaction with RBCs. Protein-NP complexes carry their own internal charge distribution that differs considerably from that of the bare NPs. Blood plasma contains proteins acting as intermediates during the interaction of cell membranes. In fact, the NPs can serve both functions, acting as enhancers or inhibitors of mediating proteins and resulting in an increase or decrease of mutual adhesion of RBCs. No change in RBC interaction is observed during incubation with polymeric NPs (see Fig. 3g), influencing neither interaction energy nor forces (see Fig. 2), thus confirming their biocompatibility.

When exposed to NDs, some RBCs change their shape to the echinocyte form (see Fig. 3h), whereas under normal conditions, RBCs are typically observed as biconcave discs (see Fig. 3a). This malformation can be associated with the formation of intercellular contacts facilitated by the surface charge of the NDs, as well as by changes in the membrane properties of the RBCs. The method of ND production and purification leads to rich unsaturated chemical bonds of carbon atoms (OH^−^, NH_2_^−^ or CO_2_H^−^ surface groups), making the surface of NDs highly reactive in its interaction with cells. The obtained results agree reasonably well with the results of alternative studies of the interaction of RBCs with carboxylated NDs (~5 nm and 100 nm in diameter) by using Raman spectroscopy and laser scanning fluorescence spectroscopy^19^. They show that smaller-sized NDs (~5 nm) induce RBC aggregation, while incubation of RBCs with larger NDs (~100 nm) causes decreased membrane deformability, making the RBCs stiffer.

In summary, the interaction of RBCs, influenced by various types of NPs, was inspected by a combined use of conventional optical microscopy, OT and SEM. While conventional optical microscopy represents a traditional method of RBC examination, the OT method contributes to the quantitative assessment of the mutual interaction of RBCs in the presence of a variety of NPs. SEM images clearly show the localization and diversity in binding of various NPs to the RBC surface. Although the localization of NPs of various sizes on the surface of RBCs takes on a multitude of forms, the results of OT measurements confirm no difference in RBC adhesion in terms of interaction energy and are in good agreement with the theoretical migration cross-bridges model. Nevertheless, an increase of adhesion forces during mutual interaction of RBCs is observed. Among the interactions with inorganic NPs, RBC incubation with NDs results in stronger RBC aggregation forces, thus leading to the formation of larger RBC aggregates and potentially influencing the shape of RBCs to which other RBCs adhere. In contrast, polymeric NPs do not influence mutual RBC interaction, so that the RBC aggregation resembles that in plasma. Thus, the results emphasize great perspectives on the combined use of OT and SEM for studying cell interaction with nanomaterials and its capability to explain observations by optical microscopy.

## Materials and Methods

### Nanoparticles

Rutile TiO_2_ RODI (Sachtleben, Germany), alumina-polyol-coated anatase TiO_2_ Hombitan AN (Kemira, Finland), uncoated anatase TiO_2_ 15 nm (Oocap, USA), uncoated ZnO (Sigma-Aldrich, Germany), carboxylated NDs (Kay Diamond, USA), and polymeric particles (synthesized as described in^28^) of different sizes (see Table 1 for details) were used in the experimental studies. The morphology of the NPs was characterized by scanning electron microscopy (SEM).

**Table 1:** Average size of the tested NPs retrieved from the size distributions obtained by SEM image analysis.

NP	Average size, nm	Surface
TiO_2_ RODI	250	rutile
TiO_2_ Hombitan AN	180	alumina-polyol coated
TiO_2_	15	uncoated anatase
ZnO	270	uncoated
Nanodiamonds	100	carboxylated
Polymeric	600	uncoated

### Red blood cells

Because no RBC aggregation occurs in phosphate buffered saline (PBS)^21^, but blood plasma proteins strongly affect RBC aggregation, the experiments were conducted in autologous plasma. To obtain platelet-free blood plasma, the blood was drawn from a clinically healthy donor by venepuncture, transferred into an ethylenediaminetetraacetic acid (EDTA)-covered vial and centrifuged at a speed of 5600 RPM (3000 g) for 10 min at room temperature (25 °C). Then, the supernatant was collected and centrifuged again under the same conditions.

Initially, prior to incubation with RBCs, all NPs were sonicated for 10 min in PBS to destroy RBC aggregates. After centrifugation, RBC pellets were accurately taken from the bottom of the vial. The obtained RBCs (8 µL) were incubated with the NP suspension in PBS (1 mL) for 1 h at room temperature (25 °C) and were centrifuged again under the same conditions (3000 g). To avoid haemolysis, the concentration of NPs incubated with RBCs was limited to 0.01% (0.1 mg per 1 mL of PBS), as a higher NP concentration caused considerable RBC shape deformation. A sufficient number of NPs were observed stuck to the membrane of RBCs, making trapping impossible and inducing RBC haemolysis in some cases. To prevent RBC aggregation and enable single-cell measurements, the RBCs with NPs were taken from the bottom of the beaker and highly diluted in plasma at a ratio of 1:100 µL (RBCs: plasma). The specimen was placed in a glass sample chamber comprising a glass slide with a coverslip attached by a double-sided adhesive tape to produce a gap of approximately 100 μm. Vaseline was applied to isolate the cuvette from the ambient air and prevent drying of the sample. The same procedure was applied to the control (NP-free) RBCs.

### Conventional optical microscopy

To estimate alterations of RBC aggregation at a multicellular level, light microscopy images were taken using an Eclipse LV100DA-U microscope (Nikon, Japan). The samples were prepared in the same cuvettes; RBCs were incubated for 2 h with NPs in the same way as described above. Then, a 3% RBC suspension was prepared using platelet-free plasma.

### Scanning electron microscopy (SEM)

The samples utilized for SEM imaging were prepared in the same way as for conventional optical microscopy and were fixed in 1% glutaraldehyde by incubation for 15 min at room temperature. Then, the samples were centrifuged at 3000 g for 10 min, and the supernatant was displaced with distilled water. A droplet of RBC suspension was dried under vacuum and covered with a 5 nm layer of platinum. SEM imaging was performed with Zeiss Ultra Plus and Sigma field emission scanning electron microscopes (Carl Zeiss, Germany).

### Statistical analysis

The results are expressed as the mean and standard deviation obtained utilizing 10 samples from the same volunteer to eliminate possible variations between RBCs and their compatibility.

### Ethics statement

All experiments were approved by the University of Oulu Ethics committee and were conducted in accordance with the ethical permission issued by the Finnish Red Cross. Informed consent was obtained from participants.

## Supplementary information

Supplementary info

## Data Availability

All data obtained and/or analysed in the frame of this study are included in this article and its Supplementary Information file.
