# Author Correction: Mutual interaction of red blood cells influenced by nanoparticles

**DOI:** 10.1038/s41598-019-44659-5

**Published:** 2019-06-05

**Authors:** Tatiana Avsievich, Alexey Popov, Alexander Bykov, Igor Meglinski

**Affiliations:** 1 Opto-Electronics and Measurement Techniques, University of Oulu, P.O. Box 4500, Oulu, 90014 Finland; 2 Interdisciplinary Laboratory of Biophotonics, National Research Tomsk State University, Tomsk, 634050 Russia; 3 National Research Nuclear University – MEPhI, Institute of Engineering Physics for Biomedicine (PhysBio), Moscow, 115409 Russia

Correction to: *Scientific Reports* 10.1038/s41598-019-41643-x, published online 26 March 2019

The Acknowledgements section in this Article is incomplete.

“The research was partially supported by EDUFI Fellowships (TM-17-10370, TM-18-10820) and the Academy of Finland (projects 290596, 314369 and 311698). IM also acknowledges partial support from the MEPhI Academic Excellence Project (Contract No. 02.a03.21.0005) and National Research Tomsk State University Academic D.I. Mendeleev Fund Program”.

should read:

“Authors acknowledge the initial involvement of Dr. M. Kinnunen (University of Oulu, Finland), Prof. A.V. Priezzhev (M.V. Lomonosov Moscow State University, Russia) and Dr. A. Karmenyan (National Don Hwa University, Hualien, Taiwan) at the early stage of the optical tweezers development and providing nanodiamonds (A.K.). The research was partially supported by EDUFI Fellowships (TM-17-10370, TM-18-10820) and the Academy of Finland (projects 290596, 314369 and 311698). IM also acknowledges partial support from the MEPhI Academic Excellence Project (Contract No. 02.a03.21.0005) and National Research Tomsk State University Academic D.I. Mendeleev Fund Program”.

