# Supplementary material for: Mutual interaction of red blood cells influenced by nanoparticles
Source: Sci Rep. 2019 Mar 26;9:5147. doi: 10.1038/s41598-019-41643-x (PMC6435805; doi:10.1038/s41598-019-41643-x)
Supplement: Supplementary file 1 — Supplementary info [file 41598_2019_41643_MOESM1_ESM.pdf]

Supplementary information

Mutual interaction of red blood cells influenced by nanoparticles

Tatiana Avsievich[1], Alexey Popov*[,1], Alexander Bykov[1], and Igor Meglinski*[,1,2,3]

[1] Opto-Electronics and Measurement Techniques Research Unit, University of Oulu, P.O. Box 4500, Oulu, 90014, Finland

[2] Interdisciplinary Laboratory of Biophotonics, National Research Tomsk State University, Tomsk, 634050, Russia

[3] National Research Nuclear University – MEPhI, Institute of Engineering Physics for Biomedicine (PhysBio), Moscow, 115409, Russia

* Correspondence: alexey.popov@oulu.fi and/or igor.meglinski@oulu.fi

Optical tweezers experimental approach

A double-channel optical tweezers (OT) experimental system (**Fig.S1**) is used for RBC manipulation at a single-cell level by two focused laser beams [1,2]. A beam from single-mode Nd:YAG infrared laser (λ = 1064 nm, max power 350 mW, ILML3IF-300 Leadlight Technology, Taiwan) is expanded by lenses L1 and L2 and then is divided into two separate beams by beam splitter BS1 to control the overall incident beam power. λ/2 waveplates and beam splitter BS2 are used for regulation of power ratio between two trapping beams. The position of the moveable trap inside the sample is controlled in the focal plane of the focusing objective by a conjugated beam adjustable mirror (M2). A water immersion objective LUMPlanFl 100× (Olympus, Japan) with a large numerical aperture (NA=1.00) allows focusing the two laser beams and forming optical traps inside the sample chamber. The sample chamber is based on the microscopy slide and cover glass separated by double-side adhesive tape (~ 100 µm thick). In the transmission mode the samples are illuminated with white LED from the bottom of sample chamber (see **Fig.S1**), and the trapped RBC are visualized by CMOS camera (Pixelink PL-B621M, Canada).

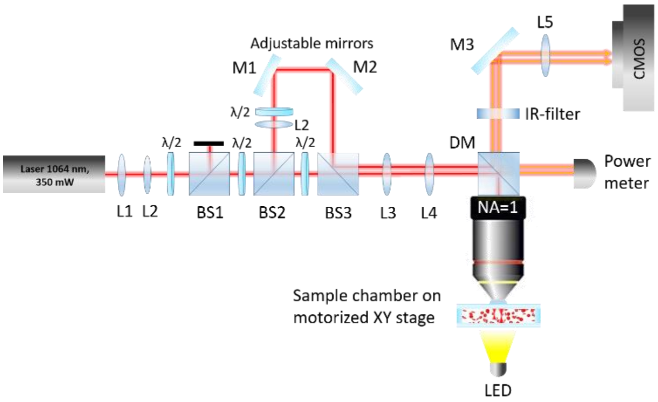

Fig. S1. Schematic presentation of the double-channel optical tweezers experimental setup. Here, L1-L5 are lenses, λ/2 are wavelength plates, BS1-BS3 are polarizing beam splitters, DM is a dichroic mirror, M1-M3 are mirrors, CMOS is a video recording camera, IR is an infrared filter.

To measure the force applied to RBC during cell trapping, calibration procedure is required. It is done by finding the maximal returning force through the matching of the trapping force with the external (viscous friction) force F_{drag} exerted on the RBC. The force of the viscous friction is calculated by the Stokes' law (defined by the force exerted on a particle in a viscous fluid):

$$F_{drag} = 6\pi\eta r v K, \tag{1}$$

where the dynamic viscosity of blood plasma at room temperature 25°C is η = 1.66×10^{-3} Pa·s [1], r is the RBC radius considering RBC to be equivolume spheres with the radius r = 2.7 µm, v is the flow velocity relative to the object, K is the correction factor defined empirically [2]. The procedure for the force calibration of each beam includes (i) trapping of one RBC with the laser beam and (ii) incremental increase of the sample chamber speed with the x-y translation stage to create a flow (shear stress). At certain speed (flow rate) the trapped RBC escapes the trap, and the interaction force is claimed to be equal to the trapping force at the given laser beam power. The heating of the trapped RBC is negligible, as 10 mW power of the laser beam focused on the cell causes temperature increase less than 1 °C.

RBC trapped with the laser beam are lifted to 30 μm height to avoid mutual interaction with other erythrocytes and the bottom of the chamber. Then, the fluid flow created by incremental increase of the speed of the moving x-y stage with the attached sample influences the RBC. The moment when RBC escapes from the trap is recorded by the CMOS video camera. The velocity of the fluid is retrieved from the video records. 10-15 RBC are used for the calibration procedure for each laser beam at different laser power values. The calibration is done for RBC incubated with NP but no difference is observed compared to untreated RBC.

RBC with the large NP aggregates (~ 1 μm) on the cell surface can be trapped with OT but the manipulation of the cell is difficult to implement in this case. The RBC-NP complex is hard to lift for interaction force measurements, arguably because the NP aggregates drastically increase the weight of RBC or change overall geometry of RBC-NP complex making the trap less efficient. Whereas, small NP aggregates (less than ~ 1 μm) localised on the RBC membrane do not influence the manipulation and/or aggregation forces in comparison to RBC samples without NP.

Aggregation force measurement

To quantify the RBC cell-to-cell interaction, the aggregation force F_a, the minimum force required to stop RBC from overlapping [3,4] is measured. Two RBC trapped by two laser beams (beam 1 and beam 2, see **Fig.S2**; power of each beam does not exceed 50 mW) are moved in contact to each other to form an aggregate with ta small interaction area (~ 3 μm in diameter); the process takes about 10 s. Then, the power of one laser beam is slowly decreased until the RBC start to overlap spontaneously by forming a doublet. We assume that the optical trapping force F (corresponding to the power of the beam losing the RBC as a result of this procedure) is equal to the interaction force between the RBC in the aggregate.

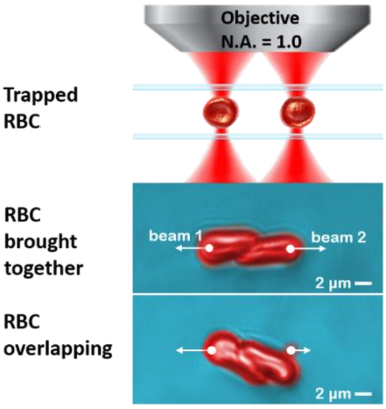

Fig. S2. Aggregation force measurement procedure. Beams 1 and 2 indicated as small white spots with arrows show opposite directions of the pulling forces of the traps.

Interaction energy calculation

The interaction energy is assessed quantitatively based on the measured interaction force as described in [4,5]. The results are presented as a function of interaction energy per conjugated surface area of interacting RBC vs. the interaction area related to the area of a single red blood cell ($\Delta S/S_{RBC}$).

References:

1. Lee, K., Danilina, A.V., Kinnunen, M., Priezzhev, A.V., and Meglinski, I., Probing the red blood cells aggregating force with optical tweezers. *IEEE Select. Topics Quantum. Electron.* **22**, 7000106 (2016).
2. Lee, K., Kinnunen, M., Khokhlova, M.D., Lyubin, E.V., Priezzhev, A.V., Meglinski, I. and Fedyanin, A.A. Optical tweezers study of red blood cell aggregation and disaggregation in plasma and protein solutions. *J. Biomed. Opt.* **21**, 35001 (2016).
3. Cooke, B.M., Stuart, J. Automated measurement of plasma viscosity by capillary viscometer. *J Clin Pathol.* **41**, 1213–1216 (1988).
4. Leith, D. Drag on nonspherical objects. *Aerosol Sci. Technol.* **6**, 153-161 (1987).
5. Avsievich, T., Popov, A., Bykov, A., Meglinski, I. Mutual interaction of red blood cells assessed by optical tweezers and SEM imaging. *Opt. Lett.* **43**(16), 3921-3924 (2018).
